# Dual-functionalized liposome by co-delivery of paclitaxel with sorafenib for synergistic antitumor efficacy and reversion of multidrug resistance

**DOI:** 10.1080/10717544.2019.1580797

**Published:** 2019-03-11

**Authors:** Meng Lei, Guanglan Ma, Sijia Sha, Xueyuan Wang, Haiting Feng, Yongqiang Zhu, Xiao Du

**Affiliations:** a College of Science, Nanjing Forestry University, Nanjing, PR China;; b College of Life Science, Nanjing Normal University, Nanjing, PR China;; c Department of Pharmaceutics, School of Pharmacy, China Pharmaceutical University, Nanjing, PR China

**Keywords:** Multidrug resistance, liposome, combination therapy, tumor targeting capability, nanomedicine

## Abstract

Multidrug resistance (MDR) remains one of the major reasons for inefficiency of many chemotherapeutic agents in cancer therapy. In this study, a D-α-tocopheryl polyethylene glycol 1000 succinate (TPGS) and polylysine-deoxycholic acid copolymer (PLL-DA) co-modified cationic liposome coating with hyaluronic acid (HA) was constructed for co-delivery of paclitaxel (PTX) and chemosensitizing agent, sorafenib (SOR) to treat the MDR cancer. The multifunctional liposome (HA-TPD-CL-PTX/SOR) presented good stability against rat plasma and was capable of reversing surface zeta potential under acidic conditions in the presence of HAase. Additionally, experimental result confirmed that the PLL-DA copolymer would facilitate the endo-lysosomal escape of the liposome. *In vitro* study demonstrated that HA-TPD-CL-PTX/SOR could significantly enhance drug accumulation in resistant MCF-7/MDR cells by inhibiting the P-gp efflux, and effectively inhibited growth of tumor cells. Furthermore, the liposome showed an enhanced anticancer activity *in vivo*, with a tumor growth inhibition rate of 78.52%. In summary, HA-TPD-CL-PTX/SOR exhibited a great potential for effective therapy of resistant cancers by combining with chemotherapeutic agents and could be a promising nano-carrier for reversing MDR and improving the effectiveness of chemotherapy.

## 1. Introduction

Cancer is still a major threat to human health. Conventional chemotherapy as the main method for the treatment of cancer has achieved no significant progress over the past 30 years. One of the well-known challenges is lack of selective accumulation in cancer cells leading to considerable damage to normal tissues (Renugalakshmi et al., 2011; Zhang et al., 2012). Another reason is the emergence of multidrug resistance (MDR), which occurred in over 50% of cases and has been a major obstacle for successful chemotherapy (Hu & Zhang, 2012). The well-studied mechanism of multidrug resistance in cancer cells is the P-glycoprotein (P-gp), an over-expression of membrane protein belonging to ATP-binding cassette (ABC) transporters, which could effectively pump anticancer agents out of cells against a concentration gradient, thereby reducing the drug concentration in the target site and eventually diminishes therapeutical efficacy (Meads et al., 2009; Fletcher et al., 2010; Yin et al., 2012). Thus, reversal of P-gp mediated chemotherapeutics efflux to overcome MDR in cancer treatment has been a promising approach.

The combination therapy has been considered as a realistic strategy for reversing drug efflux to realize successful chemotherapeutic treatment (Chang et al., 2018; Gou et al., 2018). The combination of multiple anticancer agents allows for reduction of the drug dose and provides a potential platform to simultaneously act on several anticancer targets, thereby preventing or delaying the emergence of MDR (Yang et al., 2018). However, traditional combination therapy, namely the drug cocktail, demonstrates limited success in clinics due to the non-coordinated distributions of drugs after administration (Mo et al., 2014). Additionally, the difference in solubility, potency, pharmacokinetics, and bioavailability between drugs makes the dosing schedule extremely challenging in the cocktail therapy (Lehár et al., 2009; Tai et al., 2010; Duan et al., 2013).

Nano-carriers as an interesting and effective drug delivery system have drawn many attentions for increasing drug selectivity and overcoming MDR in cancer treatment. Functional liposomes with similar lipid bilayer shell to the biofilm are one of the most investigated nanoparticle delivery systems, which possess well biological compatibility, and could increase cellular uptake and enhance intracellular drug accumulation, avoid the deficiency of inherent toxicity caused by the inhibitors, as well as bypass P-gp mediated efflux (Ji et al., 2012; Zhao et al., 2013; Tan et al., 2017). Additionally, liposomes could also play as drug reservoirs after uptake into the tumor cells, and protect drug from degradation to increase its stability. Therefore, liposomes are outstanding vesicles for co-delivery of multiple drugs based on their abilities to encapsulate both hydrophilic and hydrophobic drugs (Mo et al., 2014; Assanhou et al., 2015).

The inhibition of overexpressed P-gp would elevate the intracellular accumulation of drugs that are also P-gp substrates in general, such as paclitaxel (PTX) and doxorubicin, lead to enhanced therapeutic efficacies (Shukla et al., 2011; Yin et al., 2017). Meanwhile, sorafenib as a potent competitive multi-kinase inhibitor of the RAF/MEK/ERK signaling pathway, suppress tumor cell proliferation, survival, and angiogenesis by competitively binding to VEGFR-2, VEGFR-3, and PDGFR-b tyrosine kinases (Yang et al., 2016). The clinical trials of sorafenib exhibit a high efficacy in human hepatocellular, colon, pancreatic, breast cancer and so on (Mondal & Bennett, 2016). As reported that effective combination of sorafenib with other chemotherapeutics is a desirable approach to strengthen the effect of chemotherapy and overcome MDR in clinics (Cohen et al., 2012; Ibrahim et al., 2012; Pal et al., 2015).

**Figure 1.**
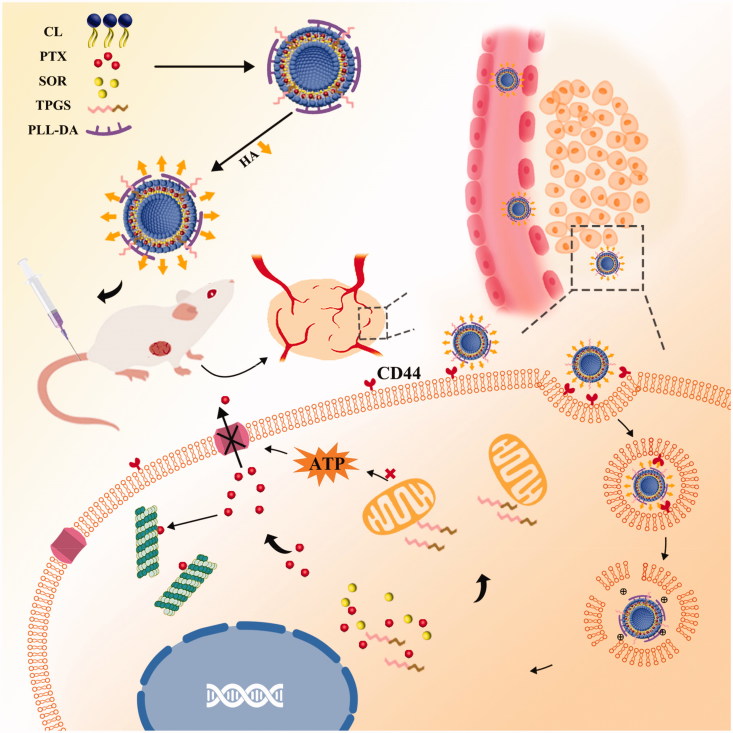
Schematic illustration of HA-TPD-CL-PTX/SOR liposome for co-delivery of PTX and SOR to overcome MDR in cancer cells. Drug delivery includes steps of intravenous injection, active targeting of liposome, degradation of HA by HAase together with the exposure of PLL-DA at HAase-rich lysosome, release of drugs, action on mitochondria function and inhibition of P-gp efflux by TPGS to further enhance drug accumulation in cancer cells.

Herein, we developed a TPGS and polylysine-deoxycholic acid (PLL-DA) co-modified cationic liposome coating with hyaluronic acid (HA) for co-delivery of paclitaxel (PTX) and sorafenib (SOR) to treat the MDR cancer. TPGS could be applied as a P-gp efflux inhibitor and solubilizer in liposome. PLL-DA embedded in the phospholipid bilayer had more primary and secondary amines that would enable carriers to facilitate endosomal escape, due to the elevation of osmotic pressure of lysosome and endosome. Furthermore, HA was applied for its biocompatibility, biodegradability, non-immunogenicity and active targeting capability (Yang et al., 2016). As illustrated in Figure 1, the multifunctional liposome (HA-TPD-CL-PTX/SOR) would preferentially accumulate at the tumor site by passive targeting effect and CD44-mediated active targeting effects after intravenous injection. Once inside of the cells, hyaluronidase (HAase) rich in tumor extracellular matrix and lysosomes would degrade the HA shell and expose the high positive charges of cationic liposome to facilitate endosomal escape and cytoplasmic distribution of the liposome. Additionally, TPGS would further interfere with mitochondrial function and block energy supply of P-gp efflux pump to minimize PTX or SOR efflux, thus maintain high therapeutic concentrations of drugs within the cancer cells and effectively reverse multidrug resistance.

## 2. Materials and methods

### 2.1. Materials

Hyaluronic acid (35 kDa) was purchased from Freda Biochem Co., Ltd. (Jinan, Shandong, China). Paclitaxel (PTX) was obtained from Shanghai Zhongxi Sunve Pharmaceutical Co., Ltd. (Shanghai, China). Sorafenib (SOR) was supplied by Meryer Chemical Technology Co., Ltd (Shanghai, China). Soybean phosphatidylcholine was provided by Shanghai Ai Wei Te Pharmaceutical Technology Co., Ltd. Cholesterol was obtained from Beijing J&L Technology Co., Ltd. Deoxycholic acid (DA) was purchased from Shanghai Macklin Biochemical Technology Co., Ltd. Triphosgene, *Nε*-carbobenzyloxy-L-lysine (Lys), hexylamine, tetrahydrofuran (THF), hexane, *N,N*-Dimethylformamide (DMF) were obtained from Energy Chemical Technology Co., Ltd (Shanghai, China).

### 2.2. Synthesis of deoxycholic acid-functionalized PLL (PLL-DA)

#### 2.2.1 Synthesis of poly (L-lysine) (PLL)

To deprotect the Z groups of PLL (Z) was dissolved in trifluoroacetic acid then HBr (2.64 g, 32.67 mmol) was added. The reaction was allowed to perform at room temperature for 2 h. Then the reaction was quenched with excess cold methyl tert-butyl ether and the precipitate was filtered and washed three times to obtain the resulting solid PLL. The average molecular weight was analyzed by gel permeation chromatography (GPC). The measurements were taken with a Shimadzu GPC with Shimadzu RI and UV/Vis detection, and two 300 mm Waters ultra-hydrogel GPC Columns using PEG standards. The dissolution solvent and mobile phase were composed of an aqueous solution containing 0.2 M NaNO_3_ and 0.01 NaH_2_PO_4_ and the mobile phase at a flow rate of 1.0 mL/min. The average molecular weight (6474 Da) and polydispersity index (1.00) were calculated from the GPC retention time (20.58 min) in Figure S2. ^1 ^H NMR (300 MHz, D_2_O, ppm): 1.22-1.86 (-CH_2_- in PLL, and -CH_2_- in hexylamine), 3.08 (dd, ε-CH_2_), 4.36 (dd, α-CH) (Figure S1).

#### 2.2.2. Synthesis of PLL-DA

The deoxycholic acid (DA) (124.31 mg, 0.32 mmol) was dissolved in 10 mL DMSO, followed by adding 1-hydroxybenzotriazole monohydrate (HOBt) (192.60 mg, 1.43 mmol), 1-ethyl-3-(3-dimethylaminopropyl) carbodiimide hydrochloride (EDCI) (273.17 mg, 0.95 mmol), respectively. Then, 0.50 g PLL and DIPEA (522.54 mg, 4.27 mmol) were added and the reaction lasted for 24 h in N_2_ atmosphere. Subsequently, the product was dialyzed against deionized water with cellulose tubing (MWCO: 3500 Da), followed by lyophilization to obtain PLL-DA.

### 2.3. Preparation of functionalized liposomes

A lipid film hydration-ultrasonic method was applied to prepare drug-loaded cationic liposome (PD-CL-PTX/SOR). Briefly, Soybean phosphatidylcholine (40 mg), cholesterol (8 mg), PLL-DA (3 mg) were dissolved in the mixture of chloroform: methanol (2:1, v:v). Then anticancer drugs PTX (1 mg) and SOR (6 mg) were added, respectively. The organic solvent was evaporated in vacuum at 45 °C to form the lipid film, which was further dried in the vacuum drying oven at 37 °C to remove residual organic solvent. The lipid film was hydrated with distilled water, and followed by homogenization at 40 °C for 1 h, which was further sonicated with a probe-type ultrasonicator in the ice bath. Then PD-CL-PTX/SOR was obtained after successive filtration 10 times through 0.22 μm membrane filters. For preparation of TPGS-functionalized liposome (TPD-CL-PTX/SOR), TPGS at the ratio of TPGS: cholesterol = 1:3 (w:w) was added into the components and dissolved in the mixture of chloroform: methanol (2:1, v:v). The following preparation method was similar to that of PD-CL-PTX/SOR. In order to realize active tumor targeting, HA was applied to coat on the surface of cationic liposome by adding TPD-CL-PTX/SOR into the HA solution at the ratio of HA: cholesterol = 3:8 (w:w) and incubating overnight at 37 °C to obtain HA-TPD-CL-PTX/SOR. Rhodamine 123 (RH123), a P-gp efflux substrate, was chosen as the fluorescent probe to monitor the cellular uptake of functionalized liposome. To prepare fluorescence labeled liposomes, drugs were replaced by RH123.

### 2.4. Physical characterization of liposomes

The morphology of multifunctional liposomes was observed by transmission electron microscopy (TEM). The particle size, zeta potential, and polydispersity (PDI) were measured via a Malvern Zetasizer 3000 system (Malvern Instruments Ltd., UK). The HPLC was used to detect the drug encapsulation efficiency (EE). Briefly, various liposomes were respectively disrupted into water and equal volume of methanol was added. Subsequently, the solution was sonicated for 20 min and the supernatant was obtained after centrifugation at 10000 rpm for 10 minutes. The amounts of PTX and SOR were measured by HPLC (Shimadzu LC-10AD system, Kyoto, Japan) coupled with a Diamonsil C18 column (250 mm × 4.6 mm, 5 μm) at a flow rate of 1 mL/min and the absorption wavelength of 227 and 266 nm. The mobile phase for PTX was methanol: water (75:25, v/v), while for SOR was a mixture of acetonitrile and disodium phosphate (buffer pH = 4 with phosphoric acid, 55:45, v/v) (Li et al., 2015). The EE was calculated using the following equations:

$$\text{EE}\left(\%\right)=\frac{\text{amount}\;\text{of}\;\text{drug}\;\;\text{in}\;\text{the}\;\text{liposome}\;}{\;\text{amount}\;\text{of}\;\text{feeding}\;\text{drug}\;}\times 100$$

### 2.5. *In vitro* drug release study

The *in vitro* release of PTX and SOR from liposome were studied by dialysis method. Briefly, 1 mL of HA-TPD-CL-PTX/SOR was sealed in a dialysis bag (MWCO: 3.5 kDa) and immersed into 30 mL PBS (incubation with or without HAase 2 mg/mL) containing 1% Tween80 (w/v) and tested at pH 7.4, or 5.0 conditions. The samples were kept at 37 °C and shaken at a speed of 100 rpm. At desired time intervals, 1 mL of release medium was taken out and equal volume of fresh media was replenished. The amount of drug released in the withdrawn medium was assessed by HPLC.

### 2.6. Stability of liposomes

The storage stability of HA-TPD-CL-PTX/SOR and PD-CL-PTX/SOR were evaluated by the change of particle size, zeta potential and drug leakage in distilled water at 4 °C for 96 h. At prearranged time (0, 12, 24, 48, 72 and 96 h), samples were withdrawn and determined. The plasma stability of above liposomes were also monitored by incubation the samples with rat plasma (1:1, v:v) and kept at 37 °C shaking with a rate of 100 rpm. At prearranged time (0, 1, 4, 8, 12 and 24 h), samples were collected and measured.

### 2.7. Cellular uptake and intracellular trafficking

The cellular uptake of different liposomes was further investigated by flow cytometry (BD, Franklin Lakes, NJ). Briefly, MCF-7 and MCF-7/MDR cells were seeded in 24-well plates at a density of 1 × 10^5^ cell/well and cultured for 24 h. Subsequently, cells were incubated with CL-RH123, PD-CL-RH123, TPD-CL-RH123 and HA-TPD-CL-RH123 for 1, 2, 4, 8, 12 and 24 h, and the fluorescence intensity was monitored by flow cytometry.

The real-time recording of the cellular internalization process of HA-TPD-CL-RH123 was assessed by confocal laser scanning microscopy (Carl Zeiss LSM 700, Germany). In brief, MCF-7/MDR cells were seeded in CLSM dish at a density of 5 × 10^5^ cells/well and cultured for 24 h. Afterward, cells were treated with HA-TPD-CL-RH123 for 1, 2, 4 and 8 h, and washed with PBS for three times. Then cells were fixed with 4% paraformaldehyde for 15 min and the cell nuclei were stained with 50 nM DAPI for 15 min. Finally, the cells were washed by PBS thrice and recorded by CLSM.

To quantitative study intracellular uptake, MCF-7/MDR cells were seeded in 6-well plates at a density of 1 × 10^6^ cells per well and cultured until a confluent monolayer of cell formed. Subsequently, different drug-loaded liposomes (PTX, 2 μg/mL) were added into each well and incubated with cells for 1, 2, 4 and 8 h, respectively. The original medium was then discarded and washed with PBS for three times. Thereafter, 150 μL of cell lysis buffer was added to fully lyse cells. The BCA Protein Assay Kit (Beyotime, China) was performed for determining the amount of protein, and the concentration of intracellular drug was detected by HPLC-MS-MS. The cellular uptake (Q_drug_/Q_protein_) was evaluated, where Q_drug_ and Q_protein_ represented the amount of drug and protein in MCF-7/MDR cells.

To further study the active targeting capability of HA-coated liposome, the CD44-overexpressing MCF-7/MDR cells were seeded in 6-well plates at a density of 1 × 10^6^ cells per well. After culturing for 24 h, the free HA (15 mg/mL) was added and incubated with cells for 2 h, followed by treatment with HA-TPD-CL-PTX/SOR for 6 h. Furthermore, the HA-coated liposome was pretreated with HAase (1 mg/mL) for 2 h, and then cells were incubated with the HAase-treated liposome for 6 h. Subsequently, the quantitative study of intracellular uptake was assessed by BCA Protein Assay Kit and analyzed by the same procedure as described above.

Confocal laser scanning microscopy (CLSM) was applied to further track the cellular transport process of different liposomes. In brief, MCF-7/MDR cells were seeded in a confocal microscope dish with 1 × 10^5^ cells/well density and cultured for 24 h. Then cells were treated with various RH123-loaded liposomes for 1 h and washed with cold PBS to remove the residual formulations. Subsequently, cells were further incubated with 1640 medium for another 0, 2 or 4 h and stained with Lyso-Tracker Red (Beyotime, China) for 90 min, and immediately imaged by CLSM.

### 2.8. Study of endocytosis pathway

To study the endocytosis pathway of HA-modified liposome, MCF-7/MDR cells were treated with specific endocytosis inhibitors including chlorpromazine hydrochloride (CH, 10 mg/mL, clathrin-mediated endocytosis inhibition), nystatin (NY, 15 μg/mL, caveolin-mediated endocytosis inhibition), amiloride (AM, 100 μg/mL, macropinocytosis inhibition) and sodium azide (SA, 3 μg/mL, ATP synthesis inhibition) for 1 h. Subsequently, HA-TPD-CL-RH123 was added to each well and incubated for another 4 h. The fluorescence intensity was determined by flow cytometry. Moreover, the viability of MCF-7/MDR cells incubated with the specific inhibitors for 1 h was also measured. All measurements were performed in triplicate.

### 2.9. In vitro cytotoxicity study

The MTT assay was used to evaluate the biocompatibility of blank liposomes and antitumor efficacy of drug-loaded liposomes. Briefly, MCF-7/MDR cells were plated into 96 well plates at a density of 5 × 10^3^ cells per well and cultured in RPMI 1640 medium for 24 h at 37 °C under 5% CO_2_ atmosphere. Subsequently, cells were incubated with different blank or drug-loaded liposomes for 48 h. MTT solution (20 μL) was added to each well and incubated for 4 h. Thereafter, the original medium was discarded and 150 μL DMSO was added. Cellular viability was calculated by measuring the absorbance at 570 nm using a microplate reader.

### 2.10. Apoptosis analysis

The cell apoptosis experiment was also evaluated by Annexin V-FITC/PI staining method. Briefly, MCF-7/MDR cells were seeded into 12-well plates until a confluent monolayer of cells formed. Then cells were treated with different drug-loaded liposomes (PTX, 1 μg/mL; SOR, 6 μg/mL) for 12 h. Afterwards, cells were collected and stained with Annexin V-FITC and PI according to the Annexin V-FITC/PI staining assay kit (Beyotime, China). At last, apoptosis-inducing capability was immediately detected flow cytometry.

### 2.11. *In vivo* antitumor efficacy

The antitumor effects were studied using BALB/c nude mice (female, 16–18 g) provided by Qinglong Mountain Animal Center and the experiment was approved by China Pharmaceutical University for the Care and Use of Laboratory Animals. The tumor model was established by injection of 0.2 mL of MCF-7/MDR cells (3 × 10^7^ cells/mice) upper the right arm of the mice. The tumor volume was calculated by (a × b^2^)/2, where a and b are the major axis and minor axis. After tumor size reached about 100 mm^3^, mice were randomly divided into six groups with five mice in each group and intravenously injected with saline, free SOR, Taxol, Taxol + SOR, TPD-CL-PTX/SOR, and HA-TPD-CL-PTX/SOR, at PTX dose of 4 mg/mg and SOR dose of 24 mg/kg. The animals were treated with the above preparations every other day (total five injections), and tumor volumes and body weight were recorded every day. Finally, mice were all sacrificed and tumors were excised, weighed and tumor inhibition rates (TIR) was calculated.

### 2.12. Statistical analysis

The data were expressed by the means ± standard deviation (SD). The statistical evaluation between different groups was analyzed by one-way ANOVA. *p* < .05 indicated statistical significance in treatment.

## 3. Results and discussion

### 3.1. Synthesis and characterization of graft copolymer

In this study, amphiphilic PLL-DA as a cationic moiety was successfully synthesized. As illustrated in Supplementary Scheme 1, Lys (Z)-NCA was synthesized using Lys (Z) with triphosgene. Subsequently, PLL (Z) was prepared through ring-opening polymerization using hexylamine as a nucleophilic initiator. PLL was obtained by successful elimination of the Z group from PLL (Z) and then reacted with carboxyl group of the deoxycholic acid (DA) by an acid-amine coupling reaction to prepare PLL-DA.

**Figure 2.**
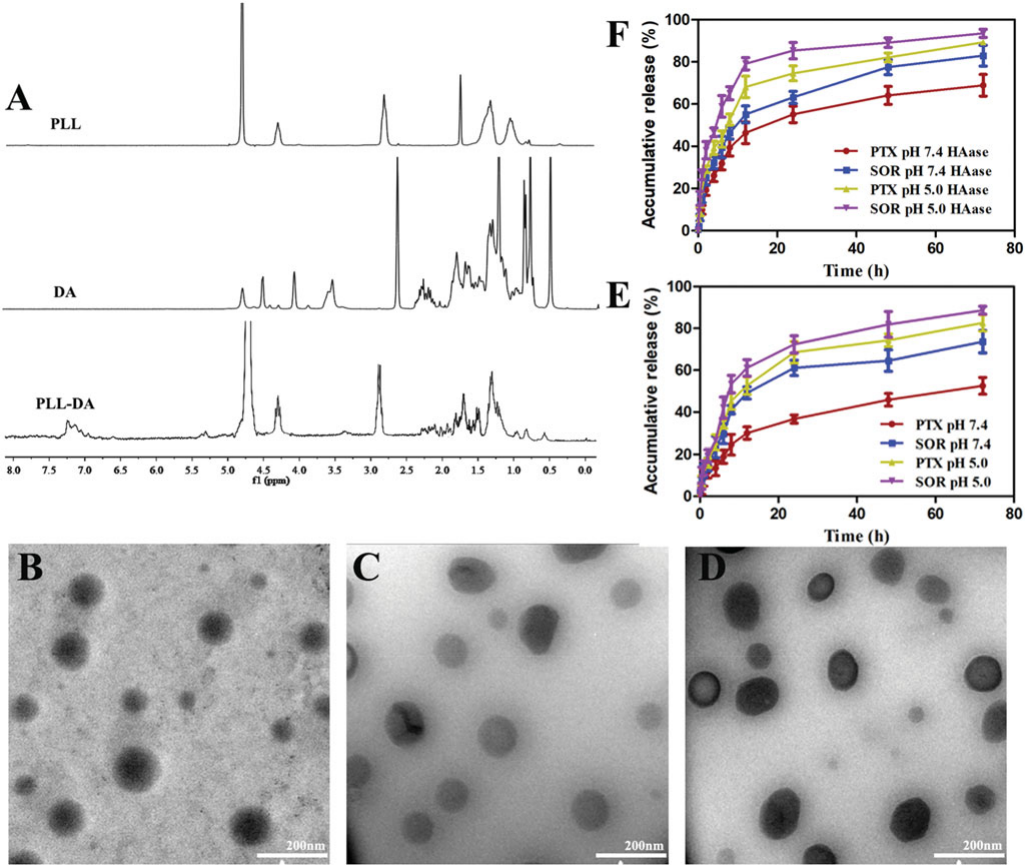
(A) ^1^H-NMR spectra of PLL, DA, and PLL-DA. TEM image of PD-CL-PTX/SOR (B), TPD-CL-PTX/SOR (C), and HA-TPD-CL-PTX/SOR (D). (E) *In vitro* drug release from HA-TPD-CL-PTX/SOR liposome at pH 7.4 and 5.0. (F) *In vitro* drug release after incubation with HAase (2 mg/mL) at pH 7.4 and 5.0 (*n* = 3).

The chemical structure of PLL and PLL-DA was confirmed by ^1^H-NMR spectra. As shown in Figure 2(A), the characteristic proton peaks at 1.10–1.72 ppm were related to methylene (-CH_2_-) and of hexylamine segment in the PLL. Signal at 2.88 ppm and 4.26 ppm was special peak of methylene of methine of PLL. The new peaks appeared in the range of 1.8–2.2 ppm for PLL-DA were ascribed to the hydrogen protons of deoxycholic acid, and 0.62, as well as 0.76 ppm, were the characteristic peaks of methyl groups of DA, indicating the successful introduction of deoxycholic acid into PLL. The substitution degree of DA in PLL was 10.87% by comparing the integral of the two peaks related to protons attributed to the methyl groups of DA at δ 0.62, with the integral related to a-carbon proton of PLL at δ 4.26 ppm (Noh et al., 2015).

### 3.2. Preparation and characterization of liposome

The cationic liposome PD-CL-PTX/SOR was prepared through a lipid film hydration-ultrasonic technique. As shown in Table S1, the zeta potential of PD-CL-PTX/SOR was approximately +31 mV, which could be attributed to the positive charge of PLL. After introducing TPGS into cationic liposome to obtain TPD-CL-PTX/SOR, the zeta potential value showed a significant reduction to +20 mV. When coating a negatively charged HA shell through electronic interaction, HA-TPD-CL-PTX/SOR exhibited a negative charge about −17 mV, which would provide sufficient repelling force between liposomes and anionic glycoproteins on the cell surface, thus avoiding aggregation between liposomes and deposition in the vessel wall (Yin et al., 2015). The particle sizes of all prepared liposomes were lower than 130 nm, which was possible to avoid filtration by the kidney, minimize specific sequestration by sinusoids in the spleen and fenestra in the liver (Duan et al., 2013). Moreover, the functionalized liposomes could efficiently entrap PTX and SOR between the hydrophobic phospholipid bimolecular layers with high encapsulation efficiency. The morphology of various liposomes was observed using TEM. As shown in Figure 2, all the liposomes were exhibited a spherical morphology and nearly mono-disperse.

### 3.3. *In vitro* drug release study

*In vitro* release kinetics of PTX and SOR-loaded liposome was tested in PBS medium at 37 °C, as displayed in Figure 2(E). The results showed a typical two-phase drug release profile, with rapid drug release in the first 12 hours, followed by subsequent slow drug release up to 72 hours. Moreover, the two drugs released from the liposomes at different release rates and the cumulative release of SOR from HA-TPD-CL-PTX/SOR was nearly 73% within 72 h in pH 7.4 condition, while that of PTX was only 52% from liposome. As pH decreased, the cumulative SOR and PTX release was all elevated to 89% and 83% in 72 h, respectively. The results illustrated that the acidic environment might be beneficial to drug release. Additionally, as shown in Figure 2(F), liposome also presented a more rapid drug release in the presence of HAase in pH 7.4 and 5.0 conditions. PTX release could increase to 68.12% within first 12 h at pH 5.0 with HAase, and the cumulative release could reach 89.33% after 72 h. In comparison, PTX release would reach 68.90% after 72 h in pH 7.4 without HAase condition. Moreover, SOR also showed a rapid release in the presence of HAase under acidic environment, and the cumulative release was 93.55%, reaching complete release by 72 h. The above results verified that drug release from liposome would be affected by pH change and HAase presence.

### 3.4. Stability of liposomes

The outstanding stability of liposome is crucial to clinical applications, including long-term storage stability in vitro and prolonged biological stability for drug targeting and circulation *in vivo* (Zhu et al., 2017). As shown in Figure S3, no significant change was observed in particle size, zeta potential and drug leakage within 96 h for HA-TPD-CL-PTX/SOR and PD-CL-PTX/SOR, indicated that the liposome could remain good stability at 4 °C for up to four days. Meanwhile, the stability of liposome under physiological conditions was also assessed using rat plasma (Figure S4). It could be observed that HA-TPD-CL-PTX/SOR also showed an obvious change in particle size and zeta potential and drug leakage in plasma for 96 h. The results illustrated that the multifunctional HA-TPD-CL-PTX/SOR could effectively maintain its structural integrity in a biological environment, which would be beneficial for its clinical applications. For comparison, PD-CL-PTX/SOR presented a slight increase in size and more drug leakage after 24 h incubation. This could be attributed to the positive charge of PD-CL-PTX/SOR that allowed it to bind to plasma protein, thereby resulting in the instability of liposome and more leakage of drugs. Thus, introduction of negatively charged HA would prevent the nonspecific protein adsorption and increased the stability of liposome.

### 3.5. Degradation of HA

HA has been reported to be closely involved in cancer metastasis, it could also improve circulation time in vivo and enhance biocompatibility and active targeting capability of nano-carriers (Yang et al., 2016). Therefore, HA was selected to mask the strong cationic surface charge of PD-CL-PTX/SOR to enhance its biocompatibility. We studied the degradation of HA by monitoring the changes in particle size and zeta potential of liposomes after incubation with HAase at different pH conditions. As shown in Figure S5, the particle size gradually increased in acidic conditions with the participation of HAase. Additionally, after incubation with HAase for 2 h at pH 7.4 condition, zeta potential of HA-coated liposome changed from −20 mV to close to neutral, while zeta potential increased from −20 mV to +15 mV at pH 5.0 condition. The results demonstrated that HA degradation was mediated by HAase and was pH-dependent. Therefore, it could be inferred that HA-modified liposome would realize charge conversion in tumor site, leading to effective intracellular delivery.

### 3.6. Cellular uptake and intracellular trafficking

**Figure 3.**
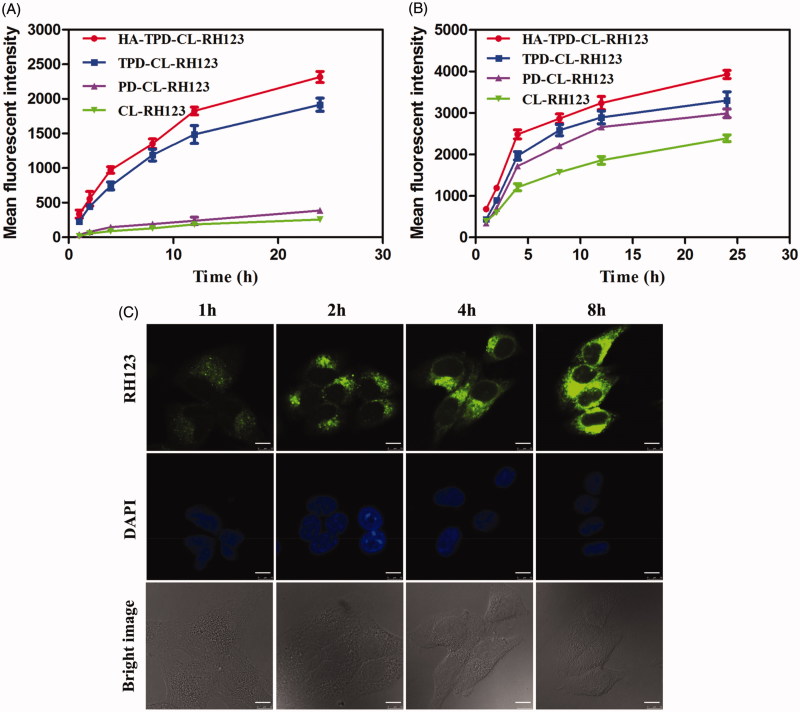
Cellular uptake of RH123 labeled liposomes in MCF-7/MDR (A) and MCF-7 cells (B). Results are expressed as mean fluorescence values determined by flow cytometry after 1, 2, 4, 8, 12 and 24 h of incubation. (C) Real-time confocal microscopy images of MCF-7/MDR after incubation with HA-TPD-CL-RH123 liposome. Scale bar: 10 μm.

Efficient cellular internalization of nano-carriers is crucial for intracellular drug delivery and efficient therapy. In order to further investigate the cell uptake of different liposomes, MCF-7 cells and MCF-7/MDR cells were incubated with CL-RH123, PD-CL-RH123, TPD-CL-RH123 and HA-TPD-CL-RH123 for 1, 2, 4, 8, 12 and 24 h, respectively, and the fluorescence signal in living cells was immediately measured by flow cytometry. As shown in Figure 3, fluorescence signal of RH123 in the two cell lines all enhanced over the incubation time, and the fluorescence intensity reached strongest at 24 h, indicating a clear time-dependent cellular uptake and effective drug delivery to cytoplasm. Moreover, the fluorescence signal of CL-RH123 and PD-CL-RH123 groups all exhibited lower fluorescence intensity in MCF-7/MDR cells, while TPGS-modified TPD-CL-RH123 and HA-TPD-CL-RH123 all displayed a significant elevation. In comparison, such a significant difference was not detected in MCF-7 cells. Thus, it was reasonable to conclude that TPGS could serve as a P-gp inhibitor for reversal of MDR and to promote the intracellular accumulation of RH123. Furthermore, compared to TPD-CL-RH123, HA-modified liposome displayed stronger fluorescence for the same interval, which could attribute to HA receptor-mediated endocytosis of the nano-carriers.

Subsequently, the real-time observation of the cellular internalization process was captured by a confocal laser scanning microscope (CLSM). As shown in Figure 3(C), the cell nuclei were stained blue with DAPI and the green fluorescence was from RH123. After 1 h of incubation, green fluorescence was obviously observed in the cytoplasm, illustrating that HA-TPD-CL-RH123 could be effective and rapid uptake into tumor cells. As the incubation period increased, green fluorescence became stronger and distributed widely in the cytoplasm, further suggesting that the cellular uptake of HA-TPD-CL-RH123 was time-independent.

**Figure 4.**
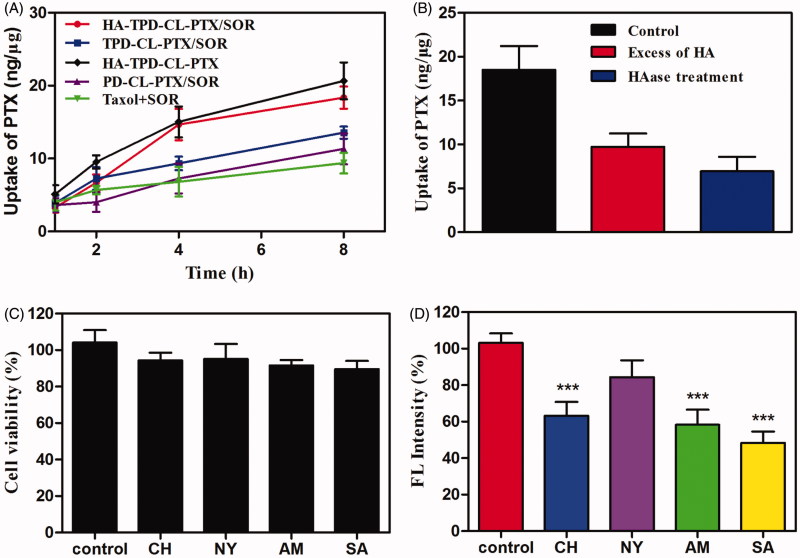
(A) Cellular uptake of PTX from different liposomes in MCF-7/MDR cells at different intervals. (B) Cellular uptake of HA-TPD-CL-PTX/SOR on MCF-7/MDR cells after 6 h in the presence of the free HA or after the HAase treatment. (C) Viability of MCF-7/MDR cells treated with different inhibitors. (D) Effects of inhibitors on endocytosis in MCF-7/MDR cells (mean ± SD, *n* = 3). Significant difference from control: ***p < .001.

The CD44-overexpressing MCF-7/MDR cells were selected to investigate cellular uptake of different drug-loaded liposomes and PTX accumulation in cells was quantified by HPLC-MS-MS. As illustrated in Figure 4(A), after 8 h of incubation, all the TPGS-modified liposomes presented a higher cellular uptake of PTX than Taxol that was even co-delivered with SOR, which could be inferred that Taxol was inevitably subject to the efflux by the overexpressed P-gp in MCF-7/MDR cells. Moreover, it was apparent that HA-modified liposomes showed a significantly higher intracellular accumulation of PTX compared to TPGS-modified liposome or the highly positive charged PD-CL-PTX/SOR, indicated that HA had played an important role in increasing intracellular uptake of liposome.

To further investigate the effect of HA on intracellular uptake, MCF-7/MDR cells were pretreated with excessive free HA and then incubation with HA-TPD-CL-PTX/SOR. As illustrated in Figure 4(B), the intracellular accumulation of PTX decreased significantly compared to the control, which could be inferred that HA-TPD-CL-PTX/SOR was ingested into cells through CD44-mediated endocytosis. Meanwhile, when incubated HA-TPD-CL-PTX/SOR with HAase at pH 4.5 for 2 h to degrade the HA shell of liposome, the intracellular uptake of the drug was further decreased, which further confirmed that HA could promote the intracellular uptake of liposome.

**Figure 5.**
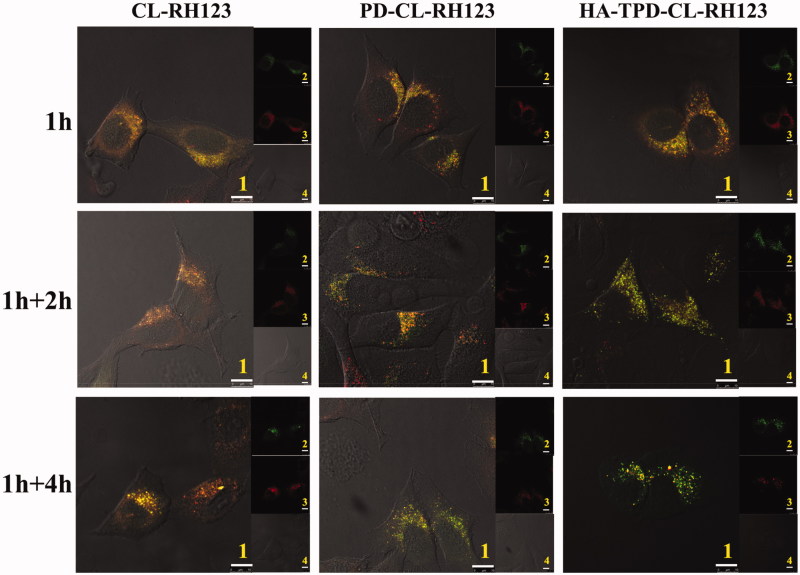
Confocal microscope images of MCF-7/MDR cells for intracellular delivery of HA-TPD-CL-RH123 for different time. The lysosomes were stained by Lyso-Tracker Red. 1: overlay of 1, 2 and 3; 2: green fluorescent of RH123-labeled liposomes; 3: red fluorescent of lysosomes; 4: bright field of cells; 1 h: incubation with liposomes for 1 h, followed by imaged by CLSM; 1 h + 2 h: incubation with liposomes for 1 h, followed by washing and further incubation for 2 h; 1 h + 4 h: incubation with liposomes for 1 h, followed by washing and further incubation for 4 h; Scale bar: 10 μm.

A major intracellular obstacle for drug delivery is endosomal entrapment followed by trafficking for lysosomal degradation or exocytosis (Zhu et al., 2018). The multifunctional liposomal carrier was applied to facilitate early endosomal escape of drug through the proton sponge effect of PLL. To investigate the intracellular trafficking and endosomal escape, CLSM was applied to observe the distribution of different RH123-loaded liposomes, and lysosomes and late endosomes were marked with Lyso-tracker Red. A yellow fluorescent signal in the merged images was observed after the colocalization of red fluorescence (Lyso-tracker) and green fluorescence (RH123-loaded liposomes), which illustrated that nano-carriers were detained by lysosomes before accumulating in cytoplasm. As illustrated in Figure 5, the co-localization of green RH123 and red Lyso-Tracker red could be detected in all treatments after 1 h of incubation, illustrating that the majority of liposomes could be entrapped into late endosomes at an early stage of cellular uptake (Yin et al., 2016). Additionally, the merged yellow signal presented no obvious change in CL-RH123 group as the incubation time increases, while PLL-modified PD-CL-RH123 and HA-TPD-CL-RH123 liposomes groups all showed significantly lower lysotracker colocalization, demonstrating success of endo/lysosomal escape.

### 3.7. Study of endocytosis pathway

Next, several specific endocytic inhibitors were applied to further investigate the endocytosis pathways of HA-TPD-CL-RH123. As shown in Figure 4(C), the cell viabilities were all above 85% after treatment with the four inhibitors for 1 h, implying that the inhibitors were nontoxic on MCF-7/MDR cells. Moreover, the inhibition of NY did not obviously change the cellular uptake of HA-TPD-CL-RH123 (Figure 4(D)). In contrast, SA remarkably decreased the cellular uptake of liposomes by about 52%, indicating HA-TPD-CL-RH123 was internalized by the cells through an energy-dependent process. Meanwhile, cell treated with CH and AM also significantly lowered the cellular uptake of RH123 by about 37% and 42%, respectively. Therefore, the results demonstrated that the multifunctional liposome was internalized by cells in an energy-dependent process, and the clathrin-mediated pathway and the macropinocytosis pathway all might play an important role in this process.

### 3.8. *In vitro* cytotoxicity study

The biocompatibility of blank liposomes was assessed against MCF-7/MDR cells by an MTT assay. As displayed in Figure S5, the cell viability was all above 80% with the concentration of HA-TPD-CL or PD-CL ranged from 0.01 to 10 μg/ml, indicated a good safety of the nano-carriers. In contrast, Taxol vehicle Cremophor EL/ethanol (1:1, v/v) showed significant cytotoxicity on MCF-7/MDR cells. Additionally, *in vitro* antitumor efficacy of drug-loaded liposomes was also investigated. As expected, HA-TPD-CL-PTX/SOR exhibited stronger cytotoxicity toward MCF-7/MDR cells than other liposomes after 48 h of incubation (Figure S5), which indicated that the HA-modified liposome could effectively trigger cargo PTX and SOR release in the tumor sites. In addition, it could be observed that the co-delivery of PTX and SOR showed an obvious decrease in tumor cell viability than HA-TPD-CL-PTX, which confirmed the effect of combination therapy. Furthermore, TPGS-functionalized liposomes all exhibited a better cell suppressive effect, indicating that TPGS potentiated capability of promoting intracellular drug accumulation and confirmed P-gp inhibiting effect of TPGS for reversal of MDR (Zhang et al., 2014; Assanhou et al., 2015).

### 3.9. Cell apoptosis

To further investigate the apoptosis-inducing effect of HA-TPD-CL-PTX/SOR on MCF-7/MDR cells, the Annexin V-FITC/PI method was applied to quantitatively analyze the apoptosis capability, and the result was assessed by flow cytometry. As presented in Figure S7, the apoptotic rate of the control group was observed to be almost negligible, while the percentage of early and late apoptotic cells was clearly increased after treatment with different preparations. The total apoptosis ratio of Taxol + SOR, CL-PTX/SOR, PD-CL-PTX/SOR, TPD-CL-PTX/SOR, and HA-TPD-CL-PTX/SOR was 49.1%, 58.8%, 63.9%, 74.0%, and 79.6%, respectively. The results demonstrate that the co-delivery of PTX and SOR liposome all showed a higher cell apoptosis effect than Taxol + SOR treatment, and this effect can be further enhanced by HA-modified liposomes, which was consistent with results obtained from the cytotoxicity study.

### 3.10. *In vivo* antitumor efficacy

**Figure 6.**
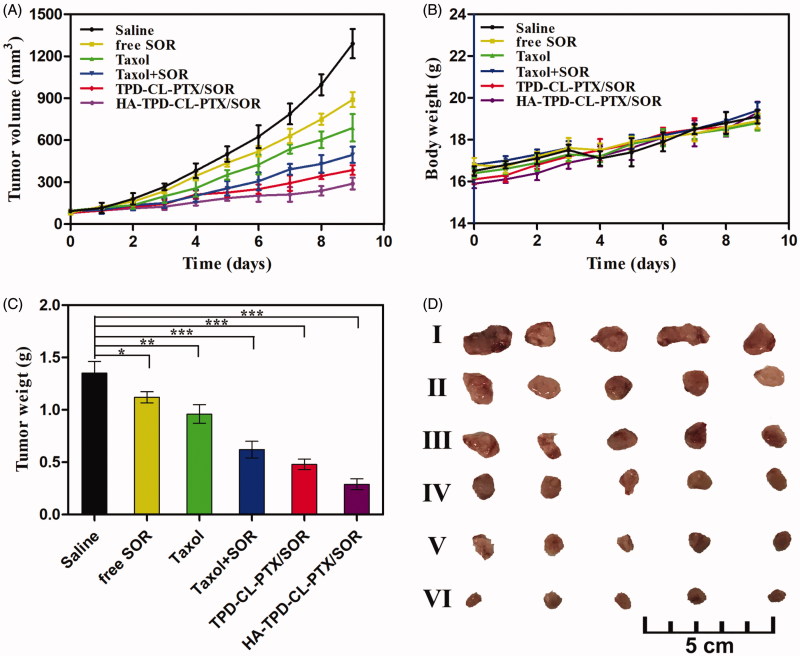
*In vivo* therapeutic efficacy of different liposomes in MCF-7/MDR tumor-bearing mice. (A) Tumor growth curves. (B) Body weight changes. (C) Average tumor weight after the study. Data were shown as mean ± SD, (*n* = 5). Significant difference from control: * *p* < .05, **p < .01, ***p < .001. (D) Images of tumor tissues treated with saline (I) free SOR (II) Taxol (III) Taxol + SOR (IV) TPD-CL-PTX/SOR (V) and HA-TPD-CL-PTX/SOR (VI).

The *in vivo* anti-tumor activity of different liposomes was investigated on MCF-7/MDR tumor-bearing mice. As shown in Figure 6(A), the tumor volume of saline group increased by 10.68-fold compared to that on the first day of treatment. Comparatively, significant tumor volume regressions were exhibited in the five formulation groups, especially HA-TPD-CL-PTX/SOR treatment. Additionally, the average tumor weight of saline group could reach to 1.35 g, while free SOR and Taxol group was 1.12 g and 0.96 g, respectively. The result showed that the free-drug was an effective tumor treatment compared with saline. Furthermore, the tumor weight of Taxol + SOR group was only 0.62 g, which was only 64.58% of the Taxol group. The superior antitumor efficacy demonstrated a synergistic effect of the two drugs. Furthermore, the TIR of free SOR, Taxol, Taxol + SOR, TPD-CL-PTX/SOR, and HA-TPD-CL-PTX/SOR was 17.04%, 28.89%, 54.07%, 64.45%, 78.52%, respectively. The superior therapeutic efficacy of HA-TPD-CL-PTX/SOR could be attributed to efficient cellular uptake, tumor-targeting of HA and TPGS co-modified nano-carriers, and the combination of multiple anticancer agents. Body weight variation was also an indicator of systemic toxicity, which was simultaneously measured every day. As shown in Figure 6(B), negligible differences were observed in the increasing body weights among all groups before and after administration, suggesting that all the liposomes did not exhibit severe systemic toxicity.

## 4. Conclusions

In summary, we developed a multifunctional liposome for co-delivery of PTX and SOR to treat the MDR cancer. This HA-TPD-CL-PTX/SOR demonstrated a series of attractive properties as an anticancer drug delivery vehicle, including ease of preparation, high loading capacity of drugs, good stability, and realizing charge conversion and endo-lysosomal escape in the tumor microenvironment, leading to release more cargo into the cytoplasm. Furthermore, the liposome showed a high drug accumulation in the tumor tissue, maintained a high intracellular drug concentration in tumor cells, and demonstrated an efficient intracellular delivery, thereby yielding the reversal of MDR and elevated cancer treatment.

## Supplementary Material

supporting_information.doc

## References

[CIT0001] Assanhou AG, Li W, Zhang L, et al. (2015). Reversal of multidrug resistance by co-delivery of paclitaxel and lonidamine using a TPGS and hyaluronic acid dual-functionalized liposome for cancer treatment. Biomaterials 73:284–95. 26426537 10.1016/j.biomaterials.2015.09.022

[CIT0002] Chang CE, Hsieh CM, Chen LC, et al. (2018). Novel application of pluronic lecithin organogels (PLOs) for local delivery of synergistic combination of docetaxel and cisplatin to improve therapeutic efficacy against ovarian cancer. Drug Deliv 25:632–43. 29463123 10.1080/10717544.2018.1440444 PMC6058476

[CIT0003] Cohen SM, Mukerji R, Timmermann BN, et al. (2012). A novel combination of withaferin A and sorafenib shows synergistic efficacy against both papillary and anaplastic thyroid cancers. Am J Surg 204:895–901. 23231932 10.1016/j.amjsurg.2012.07.027

[CIT0005] Duan X, Xiao J, Yin Q, et al. (2013). Smart pH-sensitive and temporal-controlled polymeric micelles for effective combination therapy of doxorubicin and disulfiram. ACS Nano 7:5858–69. 23734880 10.1021/nn4010796

[CIT0006] Fletcher JI, Haber M, Henderson MJ, et al. (2010). ABC transporters in cancer: more than just drug efflux pumps. Nat Rev Cancer 10:147–56. 20075923 10.1038/nrc2789

[CIT0007] Gou Y, Zhang Z, Li D, et al. (2018). HSA-based multi-target combination therapy: Regulating drugs’ release from HSA and overcoming single drug resistance in a breast cancer model. Drug Deliv 25:321–9. 29350051 10.1080/10717544.2018.1428245 PMC6058715

[CIT0009] Hu CMJ, Zhang L (2012). Nanoparticle-based combination therapy toward overcoming drug resistance in cancer. Biochem Pharmacol 83:1104–11. 22285912 10.1016/j.bcp.2012.01.008

[CIT0010] Ibrahim N, Yu Y, Walsh WR, et al. (2012). Molecular targeted therapies for cancer: sorafenib monotherapy and its combination with other therapies. Oncol Rep 27:1303–11. 22323095 10.3892/or.2012.1675

[CIT0011] Ji X, Gao Y, Chen L, et al. (2012). Nanohybrid systems of non-ionic surfactant inserting liposomes loading paclitaxel for reversal of multidrug resistance. Int J Pharm 422:390–7. 22001531 10.1016/j.ijpharm.2011.10.003

[CIT0012] Lehár J, Krueger AS, Avery W, et al. (2009). Synergistic drug combinations tend to improve therapeutically relevant selectivity. Nat Biotechnol 27:659–66. 19581876 10.1038/nbt.1549 PMC2708317

[CIT0013] Li YJ, Dong M, Kong FM, Zhou JP (2015). Folate-decorated anticancer drug and magnetic nanoparticles encapsulated polymeric carrier for liver cancer therapeutics. Int J Pharm 489:83–90. 25888801 10.1016/j.ijpharm.2015.04.028

[CIT0014] Meads MB, Gatenby RA, Dalton WS (2009). Environment-mediated drug resistance: a major contributor to minimal residual disease. Nat Rev Cancer 9:665–74. 19693095 10.1038/nrc2714

[CIT0015] Mo R, Jiang T, Gu Z (2014). Recent progress in multidrug delivery to cancer cells by liposomes. Nanomedicine (Lond) 9:1117–20. 25118703 10.2217/nnm.14.62

[CIT0016] Mondal A, Bennett LL (2016). Resveratrol enhances the efficacy of sorafenib mediated apoptosis in human breast cancer MCF7 cells through ROS, cell cycle inhibition, caspase 3 and PARP cleavage. Biomed Pharmacother 84:1906–14. 27863838 10.1016/j.biopha.2016.10.096

[CIT0017] Noh I, Kim HO, Choi J, et al. (2015). Co-delivery of paclitaxel and gemcitabine via CD44-targeting nanocarriers as a prodrug with synergistic antitumor activity against human biliary cancer. Biomaterials 53:763–74. 25890771 10.1016/j.biomaterials.2015.03.006

[CIT0018] Pal HC, Baxter RD, Hunt KM, et al. (2015). Fisetin, a phytochemical, potentiates sorafenib-induced apoptosis and abrogates tumor growth in athymic nude mice implanted with BRAF-mutated melanoma cells. Oncotarget 6:28296–311. 26299806 10.18632/oncotarget.5064 PMC4695061

[CIT0019] Renugalakshmi A, Vinothkumar TS, Kandaswamy D (2011). Nanodrug delivery systems in dentistry: a review on current status and future perspectives. Curr Drug Deliv 8:586–94. 21696348 10.2174/156720111796642336

[CIT0020] Shukla S, Ohnuma S, Ambudkar S (2011). Improving cancer chemotherapy with modulators of ABC drug transporters. Curr Drug Targets 12:621–30. 21039338 10.2174/138945011795378540 PMC3401946

[CIT0021] Tai W, Qin B, Cheng K (2010). Inhibition of breast cancer cell growth and invasiveness by dual silencing of HER-2 and VEGF. Mol Pharm 7:543–56. 20047302 10.1021/mp9002514

[CIT0022] Tan S, Zou C, Zhang W, et al. (2017). Recent developments ind-a-tocopheryl polyethylene glycol-succinate-based nanomedicine for cancer therapy. Drug Deliv 1:1831–42. 10.1080/10717544.2017.1406561 PMC8241040 29182031

[CIT0023] Yang YC, Cai J, Yin J, et al. (2016). Heparin-functionalized pluronic nanoparticles to enhance the antitumor efficacy of sorafenib in gastric cancers. Carbohydr Polym 136:782–90. 26572413 10.1016/j.carbpol.2015.09.023

[CIT0025] Yang X, Shi X, Ji J, Zhai G (2018). Development of redox-responsive theranostic nanoparticles for near-infrared fluorescence imaging-guided photodynamic/chemotherapy of tumor. Drug Deliv 25:780–96. 29542333 10.1080/10717544.2018.1451571 PMC6058498

[CIT0026] Yin M, Bao Y, Gao X, et al. (2017). Redox/pH dual-sensitive hybrid micelles for targeting delivery and overcoming multidrug resistance of cancer. J Mater Chem B 5:2964–78. 10.1039/c6tb03282f 32263989

[CIT0027] Yin S, Huai J, Chen X, et al. (2015). Intracellular delivery and antitumor effects of a redox-responsive polymeric paclitaxel conjugate based on hyaluronic acid. Acta Biomater 26:274–85. 26300335 10.1016/j.actbio.2015.08.029

[CIT0028] Yin T, Liu J, Zhao Z, et al. (2016). Smart nanoparticles with a detachable outer shell for maximized synergistic antitumor efficacy of therapeutics with varying physicochemical properties. J Control Rel 243:54–68. 10.1016/j.jconrel.2016.09.036 27702595

[CIT0029] Yin Q, Shen J, Chen L, et al. (2012). Overcoming multidrug resistance by co-delivery of Mdr-1 and survivin-targeting RNA with reduction-responsible cationic poly(β-amino esters). Biomaterials 33:6495–506. 22704597 10.1016/j.biomaterials.2012.05.039

[CIT0030] Zhang X, Guo S, Fan R, et al. (2012). Dual-functional liposome for tumor targeting and overcoming multidrug resistance in hepatocellular carcinoma cells. Biomaterials 33:7103–14. 22796159 10.1016/j.biomaterials.2012.06.048

[CIT0031] Zhang X, Li F, Guo S, et al. (2014). Biofunctionalized polymer-lipid supported mesoporous silica nanoparticles for release of chemotherapeutics in multidrug resistant cancer cells. Biomaterials 35:3650–65. 24462359 10.1016/j.biomaterials.2014.01.013

[CIT0032] Zhao YZ, Dai DD, Lu CT, et al. (2013). Epirubicin loaded with propylene glycol liposomes significantly overcomes multidrug resistance in breast cancer. Cancer Lett 330:74–83. 23186833 10.1016/j.canlet.2012.11.031

[CIT0033] Zhu Z, Li Y, Yang X, et al. (2017). The reversion of anti-cancer drug antagonism of tamoxifen and docetaxel by the hyaluronic acid-decorated polymeric nanoparticles. Pharmacol Res 126:84–96. 28734999 10.1016/j.phrs.2017.07.011

[CIT0034] Zhu J, Qiao M, Wang Q, et al. (2018). Dual-responsive polyplexes with enhanced disassembly and endosomal escape for efficient delivery of siRNA. Biomaterials 162:47–59. 29432988 10.1016/j.biomaterials.2018.01.042

