# Supplementary material for: Dual-functionalized liposome by co-delivery of paclitaxel with sorafenib for synergistic antitumor efficacy and reversion of multidrug resistance
Source: Drug Deliv. 2019 Mar 11;26(1):262–72. doi: 10.1080/10717544.2019.1580797 (PMC6419656; doi:10.1080/10717544.2019.1580797)
Supplement: supporting_information.doc [file IDRD_A_1580797_SM7567.doc]

**Part A: Experimental Section**

**1. Synthesis of *Nε*-carbobenzyloxy-L-lysine N-carboxyanhydrides (Lys(Z)-NCA)**

The Lys(Z)-NCA was synthesized as previous reported and had slightly modification (Noh et al., 2015). Briefly, Lys(Z) (2.00 g, 7.14 mmol) was dissolved in the 30 mL THF and then triphosgen (0.42 g, 1.43 mmol) was added. After reaction at 50°C for 3 h, the solvent was evaporated and the crude product washed with 50 mL of [hexane](javascript:showMsgDetail('ProductSynonyms.aspx?CBNumber=CB1852811&postData3=CN&SYMBOL_Type=A');). The purified product was then dried in a vacuum oven. ^1^H NMR (300 MHz, DMSO-d_6_, ppm): δ 1.21-1.52 (m, γ-CH_2_ and δ-CH_2_), 1.71 (ddd, β-CH_2_), 2.97 (q, ε-CH_2_), 4.36 (t, α-CH_2_), 5.02 (s, -CH_2_- in Z groups), 7.20-7.41 (m, -Ph in Z groups) (Fig. S1).

**2. Synthesis of poly (*Nε*-carbobenzyloxy-L-lysine) (PLL (Z))**

Lys(Z)-NCA (2.80 g, 9.14 mmol) was dissolved in the 20 mL DMF and [hexylamine](javascript:showMsgDetail('ProductSynonyms.aspx?CBNumber=CB4489344&postData3=CN&SYMBOL_Type=A');) (7.33 mg, 0.07 mmol) in 1 mL DMF was added. The reaction was allowed to stir at 35°C for 48 h at N_2_ atmosphere. Then the reaction was poured into the mixture of cold methyl tert-butyl ether and [hexane](javascript:showMsgDetail('ProductSynonyms.aspx?CBNumber=CB1852811&postData3=CN&SYMBOL_Type=A');). The precipitate was filtered, dried in vacuum and yield as PLL (Z). ^1^H NMR (300 MHz, DMSO-d_6_, ppm): 4.98 (s, -CH_2_- in Z groups), 7.20-7.45 (m, -Ph in Z groups) (Fig. S1).

**3. Degradation of HA**

The HAase-mediated degradation of HA was assessed by the change in particle size and zeta potential of HA-coated liposome at pH 5.0 and pH 7.4. In brief, the mixture of HA-TPD-CL-PTX/SOR and HAase (2 mg/mL) was placed in the 37°C water bath. At predetermined time the particle size and zeta potential was measured.

**4. The inhibitory effects on P-gp mediated drug efflux**

RH123 as a P-gp efflux substrate was applied to evaluate the effect of TPGS on reversal of MDR phenomenon. Briefly, MCF-7 and MCF-7/MDR cells were seeded in 24-well plates until a confluent monolayer of cell formed and then treated with different concentration of TPGS for 1, 2 and 4 h, respectively. Subsequently, cells were washed with cold PBS and further incubated with 1640 medium containing RH123 (5 μM) for 2 h before observation by a fluorescence microscope (Olympus IX 51, Osaka, Japan).

**Part B: Results**


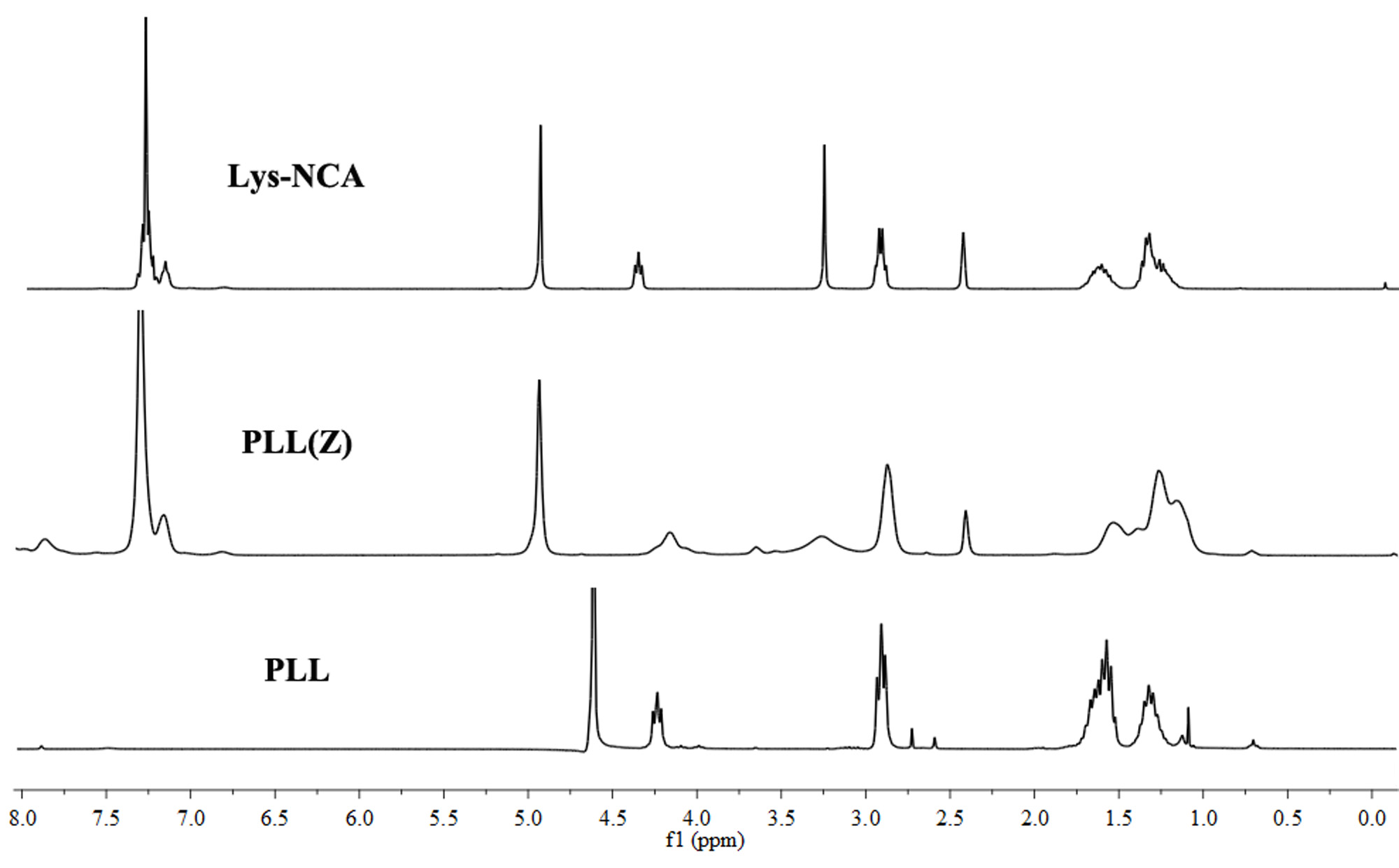


**Fig. S1.** ^1^H-NMR spectra of Lys(Z)-NCA, PLL (Z), and PLL.


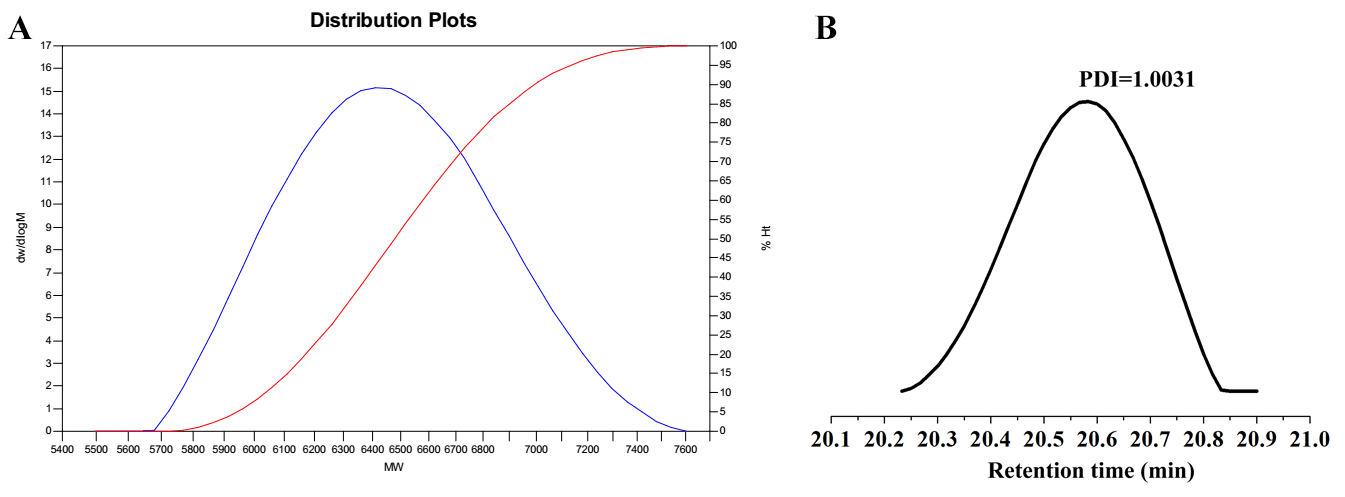


**Fig. S2.** (A) The average molecular weight (Mw) of PLL(Z) and the (B) GPC retention time was measured by gel permeation chromatograph (GPC).


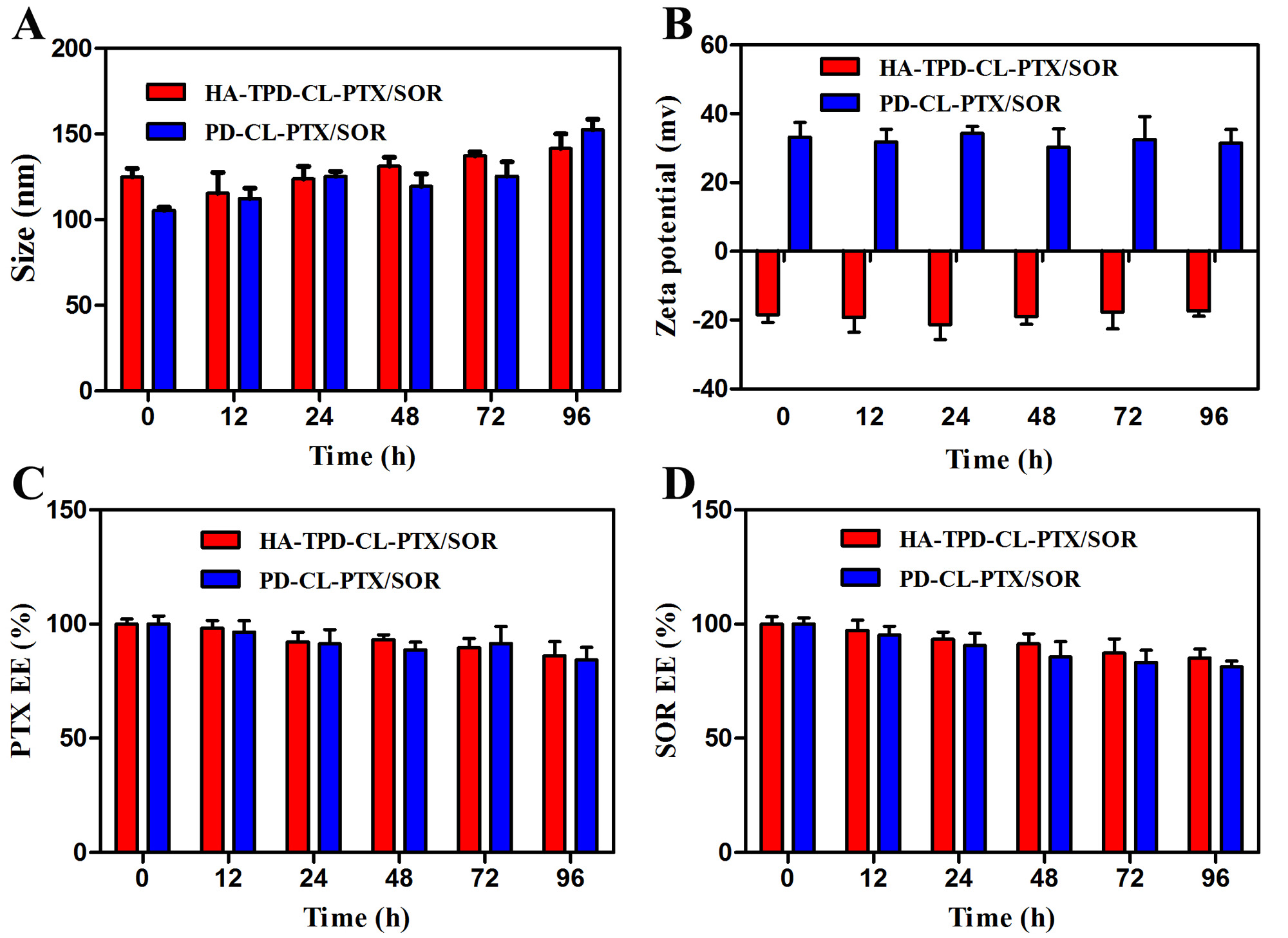


**Fig. S3**. Changes of particle size (A), zeta potential (B), and entrapment efficiency (EE) of PTX (C) and SOR (D) of different liposomes after storage at 4°C for 4 days (mean±SD, n=3).


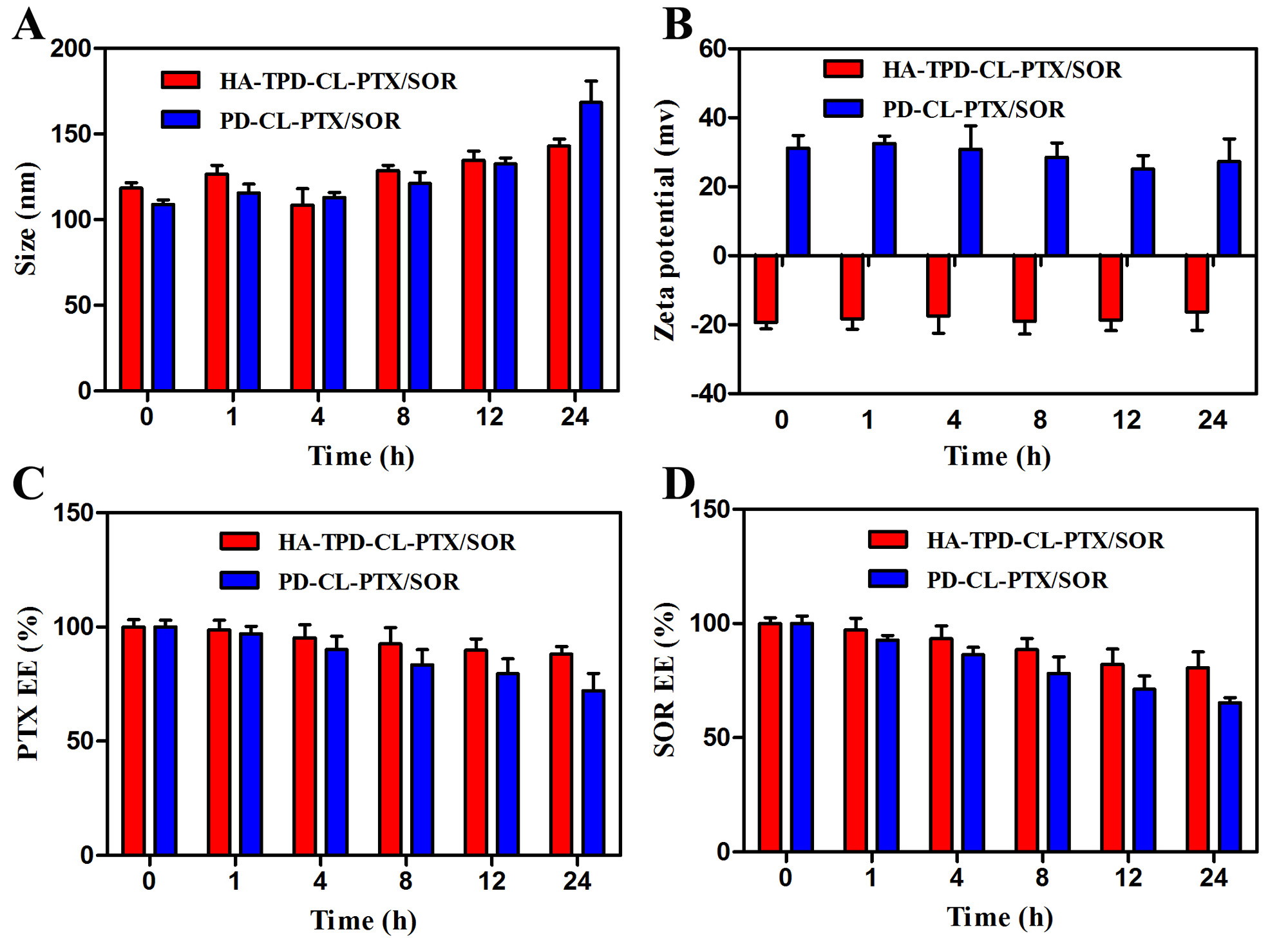


**Fig. S4**. Changes of particle size (A), zeta potential (B), and entrapment efficiency (EE) of PTX (C) and SOR (D) of different liposomes after incubation with plasma over time (mean±SD, n=3).


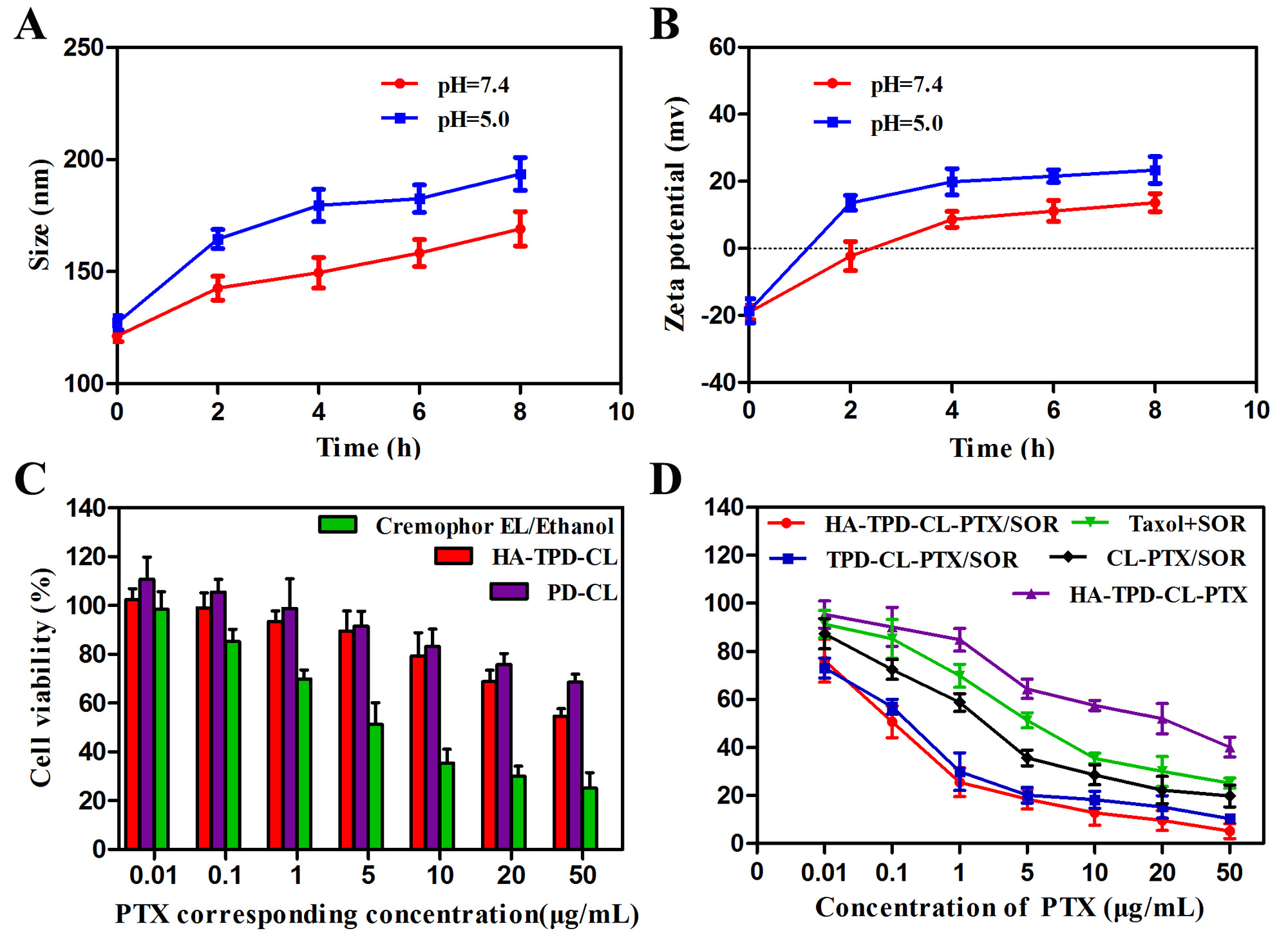


**Fig. S5**. (A) Changes in particle size and (B) zeta potential of HA-TPD-CL-PTX/SOR liposome after incubation with HAase (2 mg/mL) at pH 7.4 and 5.0. In vitro cytotoxicity of blank vehicles (C) and different liposomes (D) against MCF-7/MDR cells at different concentrations after 48 h incubation (mean±SD, n = 6).

The overexpressed P-gp pump on the cell membrane of the MDR cancer cells played an important role in the efflux of the anticancer drugs, which would lead to a low intracellular drug concentration. Thus, we further studied the effects of TPGS on intracellular uptake on MCF-7/MDR cells by fluorescence microscope. RH123 as a P-gp efflux substrate was applied to act as a model drug. As illustrated in Fig. S6, MCF-7 cells presented a higher fluorescence intensity after treatment with free RH123 for different time, but hardly entered into MCF-7/MDR cells, which demonstrated a strong P-gp-mediated efflux of RH123 by MCF-7/MDR cells. In contrast, after treated with different concentrations of TPGS, fluorescence intensity increased significantly in MCF-7/MDR cells, which further confirmed the P-gp inhibiting effect of TPGS for reversal of MDR.


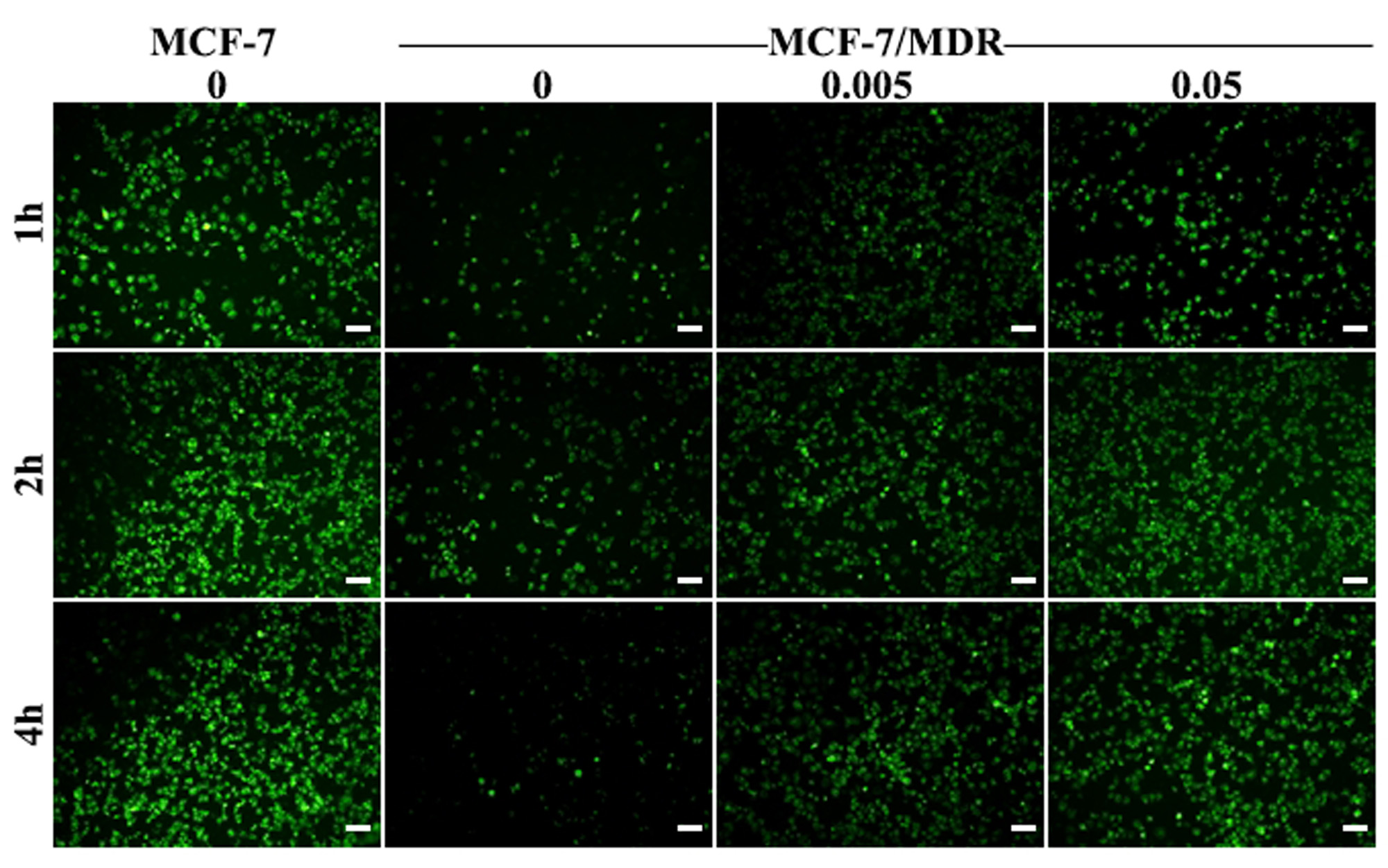


**Fig. S6**. Fluorescence images of MCF-7 and MCF-7/MDR cells after 1 h of incubation with the free RH123 in absence and presence of different concentrations of free TPGS. Scale bar: 50 μm.


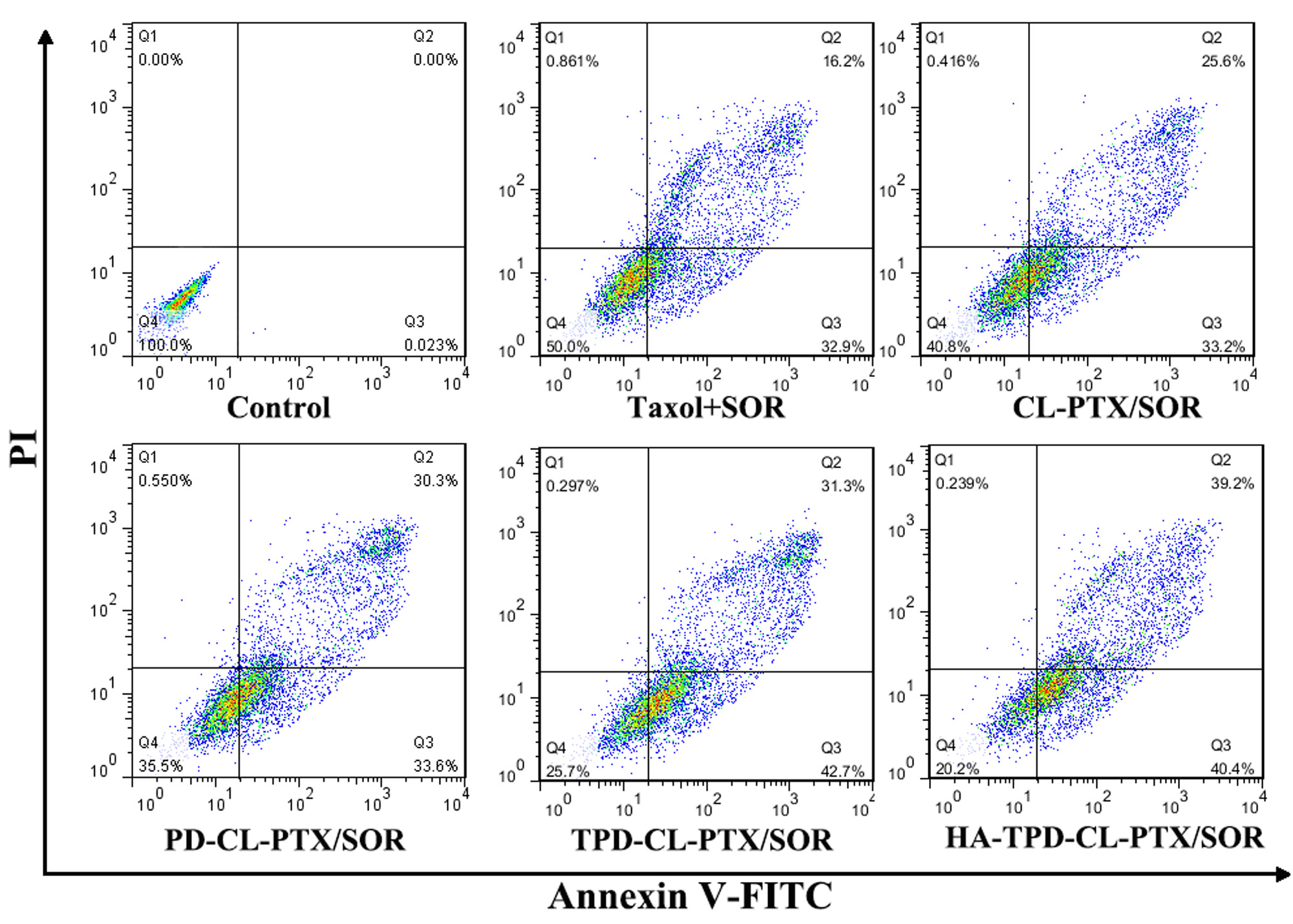


**Fig. S7**. Apoptosis rate of MCF-7/MDR cells was determined by Annexin V-FITC/PI staining. The lower-left, lower-right, upper-right, and upper left quadrants represented the viable, early apoptotic, late apoptotic and dead cells, respectively.

**Table S1 Characteristics of different drug-loaded liposomes (n=3)**

| Liposome | Particle size (nm) | PDI | Zeta (mV) | EE (%) | |
| --- | --- | --- | --- | --- | --- |
|  |  |  |  | PTX | SOR |
| CL-PTX/SOR | 127.44 ±2.65 | 0.26 | -22.85 ± 2.7 | 90.38 ± 2.7 | 85.56 ± 4.9 |
| PD-CL-PTX/SOR | 109.61 ±3.18 | 0.19 | +31.43 ± 3.5 | 83.14 ± 4.6 | 82.64 ± 1.7 |
| TPD-CL-PTX/SOR | 94.11 ± 1.95 | 0.22 | +19.61 ± 1.8 | 88.51 ± 2.1 | 87.97 ± 3.3 |
| HA-TPD-CL-PTX/SOR | 98.72 ± 4.29 | 0.17 | -16.83 ± 3.9 | 85.26 ± 3.7 | 83.22 ± 1.2 |

**Scheme.1** Synthetic routes of the graft copolymer PLL-DA.
